# Extending the Implicit Association Test (IAT): Assessing Consumer Attitudes Based on Multi-Dimensional Implicit Associations

**DOI:** 10.1371/journal.pone.0015849

**Published:** 2011-01-05

**Authors:** Valentin Gattol, Maria Sääksjärvi, Claus-Christian Carbon

**Affiliations:** 1 Department of Product Innovation Management, Faculty of Industrial Design Engineering, Delft University of Technology, Delft, The Netherlands; 2 Department of General Psychology and Methodology, University of Bamberg, Bamberg, Germany; Rand, United States of America

## Abstract

**Background:**

The authors present a procedural extension of the popular Implicit Association Test (IAT; [1]) that allows for indirect measurement of attitudes on multiple dimensions (e.g., safe–unsafe; young–old; innovative–conventional, etc.) rather than on a single evaluative dimension only (e.g., good–bad).

**Methodology/Principal Findings:**

In two within-subjects studies, attitudes toward three automobile brands were measured on six attribute dimensions. Emphasis was placed on evaluating the methodological appropriateness of the new procedure, providing strong evidence for its reliability, validity, and sensitivity.

**Conclusions/Significance:**

This new procedure yields detailed information on the multifaceted nature of brand associations that can add up to a more abstract overall attitude. Just as the IAT, its *multi-dimensional* extension/application (dubbed *md*-IAT) is suited for reliably measuring attitudes consumers may not be consciously aware of, able to express, or willing to share with the researcher [2], [3].

## Introduction

Traditionally, attitudes have been measured by having consumers respond to an attitude object (or entity) on self-report rating scales. In these scales, consumers rate a particular object (e.g., a product or a brand) on dimensions such as “good/bad”, “like/dislike”, or “pleasant/unpleasant”. Yet, consumers often find it difficult to report on these scales. They may not have attitudes readily available for reporting on them (in an explicit way), or may even find it difficult to retrieve them [2], [3]. Indirect measures, in particular the popular Implicit Association Test (IAT) by Greenwald, McGhee, and Schwartz [1], constitute a viable alternative avoiding some of the problems associated with direct measures (e.g., lack of attitude availability/accessibility, social desirability bias). In this article, we introduce a procedural extension of the IAT, a *multi-dimensional* Implicit Association Test (*md*-IAT). In contrast to the regular IAT, which is utilized as a procedure that allows assessment on a single dimension only, the *md*-IAT comprises six dimensions, thus allowing for a more detailed, multi-dimensional assessment of attitudes. More fine-grained attitudes/associations have been assessed in several studies but were confined to a single administration and thereby also to a single dimension in the IAT: for example, to measure gender stereotypes (i.e., men–women/warm–cold [4]), self-concepts (i.e., self–other/anxious–calm, [5]), or even abnormal pedophilic tendencies (i.e., children–adults/sex–no-sex, [6]). The additional information offered by this multi-dimensional measure can be of particular value in marketing and consumer research, allowing for example—in the same way as with direct measures—to easily create more complex and differentiated profiles of products and brands (cf. [7]). Tapping consumer insights in such a way more appropriately captures the richness of consumers' perceptions, feelings, and attitudes toward a brand. For example, the IAT can indeed provide important information about consumers' general attitude toward a specific brand or product (consumers' likes and dislikes), but it does not elucidate the different components contributing to this global attitude. Any kind of intervention, however, depends on clear diagnostics: the specific aspects consumers like or dislike or the specific properties they associate with the product [8]. The contribution of the present research is both of theoretical and practical relevance: our results show that the *md*-IAT procedure is a methodologically sound extension of the IAT that—unlike the latter—also allows for multi-dimensional assessment of brand attitudes. This in turn opens up numerous possibilities for researchers to test constructs such as brand or product personality [9], [10], or more generally, consumers' brand associations or attitudes on any kind of multi-dimensional scale [11]. In addition, we show that the *md*-IAT, just like the IAT, is not affected by the specific stimuli selected to represent a brand. The three brand identifiers used in the present studies (logos, signatures, and product pictures) all yielded similar results, therefore rendering the *md*-IAT rather suited as a conceptual (as opposed to perceptual) measure of brand attitudes.

The structure of the paper is as follows: We start by briefly reviewing different forms of attitude measurement—distinguishing between indirect and direct measures. We then turn to the IAT itself before introducing its *multi-dimensional* extension (the *md*-IAT) and its application in two within-subjects repeated measurement studies.

### Indirect versus Direct Measures

Indirect measures differ from direct measures in that they do not rely on verbal self-reports as a way of inferring attitudes [12]. Instead, they rely on rather indirect means of assessing an attitude, for example differences in reaction times, facial expression, or specific brain activation. Indirect measures can be further distinguished into physiological or latency based measures. Physiological measures include techniques such as electro-dermal activity (EDA; [13]), pupillometry [14], eyetracking [15], electromyography (EMG; [16]); or various brain imaging techniques, such as functional magnetic resonance imagining (fMRI; [17]), which allow direct observation of brain activity during mental tasks. While promising in their own right, these physiological measures do not yet offer standardized forms of attitude assessment (for advances in this domain, see, [16], [18]). In addition, they require (very) expensive equipment and a considerable expertise in the domain of cognitive neuroscience, which make most of these research techniques inaccessible and/or ill-suited for any kind of more applied research. This is much less the case for indirect measures based on response latencies (or reaction times). Measures such as affective priming [19], the Extrinsic Affective Simon Task [20], the Go/No-Go Association Task [21], and particularly the Implicit Association Test (IAT; [1]), are fairly standardized forms of attitude assessment requiring little more than a computer and a testing environment void of external distractions.

### Attitude Measurement and the Implicit Association Test (IAT)

The IAT is a method of estimating evaluative associations that underlie implicit attitudes, which draws on differences in reaction times in a rapid computerized categorization task. Introduced more than a decade ago by Greenwald, McGhee, and Schwartz [1], it is one of the most widely used indirect attitude measures. The IAT is considered superior to the other latency-based techniques mentioned above, showing moderate-to-high correlations with self-report attitude measures in the consumer domain [2], [22]–[26] and satisfactory split-half reliabilities [22], [27], [28]. The IAT has also shown to be quite robust with regard to stimulus artifacts. That is, stimulus specifics, for example in the visual domain, seem to be of little importance as long as category membership remains unambiguous [12], [29]. Brunel et al. [2] tested the applicability of the IAT in consumer research and concluded that the IAT is a valid measurement instrument for capturing consumer attitudes. In two studies, they showed that the IAT was sensitive to individual differences in attitude accessibility and that the IAT can capture automatic associations that are distinct from explicit measures.

#### Conscious and Less Conscious Manifestations of Attitudes

Up until the late 1990s research in the domain of attitudes largely involved assessing attitudes by means of direct measures. Direct measures require participants to consciously or deliberately think about a certain attitude object and subsequently report their attitudes in the form of verbal self-reports, for example, on semantic differential scales or Likert-scales [30]–[32]. By means of such explicit introspective processing, participants arrive at an attitude toward an object, either by retrieving it from memory or by constructing it on the spot. In contrast, indirect measures try to measure participants' implicit attitudes, which Greenwald and Banaji [33] describe as “introspectively unidentified (or inaccurately identified) traces of past experience that mediate favorable or unfavorable feeling, thought, or action toward social objects” (p. 8). Greenwald and Banaji introduced this implicit–explicit dichotomy to attitude research. Since then the implicit-explicit terminology has become popular for referring both to the form of measurement (indirect vs. direct) and the form of representation in memory (unconscious vs. conscious). Greenwald and Banaji note that attitudes—in addition to their conscious manifestations—might also operate in an indirect, unconscious, or implicit mode. Such implicit attitudes are activated automatically, not necessarily requiring conscious thought or attention [19]. Whether or not implicitly measured attitudes are also (truly) unconscious is widely debated since participants might be unaware that their attitudes are being assessed, but this does not necessarily imply unawareness of possessing those attitudes [12], [34]. Despite these reservations, indirect measures seem particularly useful when consumers do not have readily available attitudes that they could consciously report on—attitudes consumers may not be aware of, able to express, or willing to share with the researcher [2], [3].

#### Design of the IAT

The IAT has shown to be a flexible and fairly easy-to-use tool in assessing strengths of associations between different concepts, contributing notably to its attractiveness and widespread use in research [27]. Typically, the IAT engages subjects into a sorting task requiring them to quickly sort stimuli (e.g., pictures or words) into one of four categories. The categories themselves are referred to as target categories and attribute categories; for example, in an IAT assessing cultural stereotypes and prejudice, one could employ the categories “American” and “European”, “pleasant” and “unpleasant”, respectively. The category names are displayed in the top corners of the computer screen, whereas the stimuli (e.g., pictures of famous Americans/Europeans and words with a clear pleasant/unpleasant connotation) appear in the center. The IAT comprises five consecutive tasks: the target discrimination task (task 1), the attribute discrimination task (task 2), the initial combined task (task 3), the reversed target discrimination task (task 4), and the reversed combined task (task 5). Throughout tasks 1–5 subjects respond by pressing either one of two keys; that is, the “left key” for stimuli belonging to a category on the left side of the screen, and the “right key” for stimuli belonging to a category on the right side of the screen. The first two tasks are intended to familiarize the subjects with both the stimuli and the overall assignment. Subjects are either required to sort target category stimuli to the target categories (task 1) or attribute category stimuli to the attribute categories (task 2). Unlike tasks 1, 2, and 4, which assign each key to only one category, the combined tasks assign each key to two categories. Referring to our example, “American” and “pleasant” might be assigned to the “left key” for the first combined task, requiring “European” and “unpleasant” to be assigned to the “right key” (or vice versa). The second combined task is identical to the initial combined task, except for the target categories (i.e., “American” and “European”) being reversed. Due to the change in target categories, subjects need to unlearn the previous key assignments and rehearse the new key assignments in an intermediate task (task 4). The dependent measure (i.e., the “IAT-effect”) is calculated as a difference score by subtracting the average response time of the initial combined task from the average response time of the reversed combined task. A positive IAT-effect is interpreted as a stronger association for the category pairing in the initial combined task—for attitude-IATs it may as well be interpreted as a preference for one concept over the other [1].

IAT scripts are usually based on a seven-block (seven-task) structure. This is because earlier research essentially employed a seven-task model in which each of the combined tasks was preceded by a combined task practice block that was shorter but otherwise identical. Originally, these preceding practice blocks were not used for computing the IAT-effect. Although Greenwald, Nosek, and Banaji [35] proposed a new scoring algorithm, the *D measure*, which draws also on data from the combined practice blocks for computing the IAT-effect, most scripts for analyzing IAT-effects still use traditional routines for dividing the analysis into seven blocks.

### The *multi-dimensional* Implicit Association Test (*md*-IAT)

The main idea behind the present research was to extend the IAT procedure to allow for a valid *multi-dimensional* assessment of attitudes that is also economically feasible (i.e., the diagnostic value in proportion to the time and effort invested). Instead of employing just one IAT, using for example *good–bad* as the single attribute dimension (as typical for attitude-IATs), the *multi-dimensional* Implicit Association Test (*md*-IAT) consists of several IATs, each aimed at measuring different aspects of a more abstract, general attitude. Most definitions of attitudes consider affective-evaluative components to be most essential in attitudes. Attitude measures typically ask participants to evaluate an attitude object along attribute dimensions such as good–bad or favorable–unfavorable [31], [36]. By having participants evaluate two target concepts (in our case automobile brands) on several distinct attribute dimensions rather than just a single overall attribute dimension, it is possible to obtain a more detailed and differentiated account of consumers' associations with a brand, similar to that of brand (personality) profiles generated by semantic differential scales known from the tradition of explicit measures.

Naturally, in introducing a new measure or—as in this case—an extension to an existing measure, it is important to address its methodological appropriateness. Reliability of the *md*-IAT was assessed by calculating the IAT-effects separately for odd and even trials and correlating these two scores (for each IAT in the *md*-IAT procedure) using a Spearman-Brown correction (see [22]). Of particular interest was whether participants could handle six IATs in a row, that is, whether the *md*-IAT, despite requiring multiple administrations, would preserve the same level of reliability. Validity of the *md*-IAT was assessed in two ways: First, by comparing the results from the IATs to direct (or explicit) ratings of the same six attribute dimensions; and second, by adding the factor *brand cue*, which involved brand stimuli varying by their level of abstraction. Based on previous findings that identified the IAT to be more driven by the target category labels than by the actual stimuli in the sorting task [12], [27], [29], differences due to this factor were not expected. Obtaining similar results, regardless of the brand cue used, may thus be interpreted as evidence for its external validity—making the *md*-IAT better suited for conceptual brand assessment and less prone to idiosyncrasies in the perceptual domain.

Thus, compared to a regular IAT, the main benefit of the *md*-IAT lies in its more detailed and differentiated assessment of consumers' brand attitudes. With such a method in hand, practitioners can easily create brand profiles based on indirect measures that provide more information than simply how *good or bad* a brand is. This, in turn, will also provide more opportunities for specific intervention in practice. In this article, we draw on the results of two within-subjects repeated-measurement studies to provide evidence both for the methodological appropriateness and practical utility of the extended, *multi-dimensional* IAT procedure.

## Materials and Methods

### Study 1

#### Participants

Thirty volunteers (15 women) participated in the study. The sample consisted of adults from the Vienna Metropolitan Area, both students and young professionals between the ages of 20–40 (median age  =  27.0 years). Two female subjects were excluded prior to the analysis after reporting difficulties with the task upon debriefing. An additional two subjects (one male, one female) were excluded after the analysis of the reaction time data because of an average total error rate of more than 10% across all IATs. Among the remaining participants, 76.9% (20) were car owners. The average overall interest in cars showed to be low among the participants (*M* = 2.0, *SD* = 1.93). Overall interest in cars was assessed by six yes–no questions (“I buy and read car magazines”; “I watch broadcasts about cars on TV”; “I am interested in cars”; “I actively follow the latest developments in the car sector”; “I talk about different car models with friends and/or family members”; “I pay attention to car advertisements”) which were then summed up to form an index (range 0–6). All subjects had normal or corrected-to-normal vision (visual acuity was checked with standard Snellen charts).

#### Materials

The present research was interested in indirectly assessing participants‘ brand attitudes toward two automobile brands using a multi-dimensional extension of the IAT, the *multi-dimensional*-IAT (*md*-IAT), as the dependent measure. Instead of employing just one IAT and therefore only one attribute dimension (e.g., *pleasant–unpleasant*), the present research was based on a more complex design that involved administering six consecutive IATs, each intended to measure associations on a different attribute dimension. The six bipolar attribute dimensions were selected on the basis of highly relevant properties derived from consumer research [7], [10], [37]–[39]: (1) safe–unsafe (2) young–old (3) reliable–unreliable (4) aggressive–peaceful (5) environmentally friendly–non-environmentally friendly (6) innovative–conventional. Each pole (or attribute category) was represented by three word stimuli (see table 1 for a complete list of the word stimuli used in all of the IATs).

**Table 1:** Word stimuli for each category of the six bipolar attribute dimensions, translated into English (original German terms used in the study are given in parentheses).

DIMENSION	ATTRIBUTE CATEGORY 1	ATTRIBUTE CATEGORY 2
1	safe (sicher)Switzerland (Schweiz)airbag (Airbag)	unsafe (unsicher)Iraq (Irak)dangerous (gefährlich)
2	young (jung)child (Kind)junior (Junior)	old (alt)grandpa (Opa)senior (Senior)
3	reliable (zuverlässig)measurement (Messung)dependable (verlässlich)	unreliable (unzuverlässig)estimation (Schätzung)non-dependable (unverlässlich)
4	aggressive (aggressive)Rottweiler (Rottweiler)Rambo (Rambo)	peaceful (friedlich)rabbit (Kaninchen)Gandhi (Gandhi)
5	environmental (ökologisch)bicycle (Fahrrad)recycling (Recycling)	non-environmental (unökologisch)motorcycle (Motorrad)toxic waste (Giftmüll)
6	innovative (innovativ)progress (Fortschritt)ICE-train (ICE-Zug)	conventional (konventionell)standstill (Stillstand)steam train (Dampflok)

**Figure 1:**
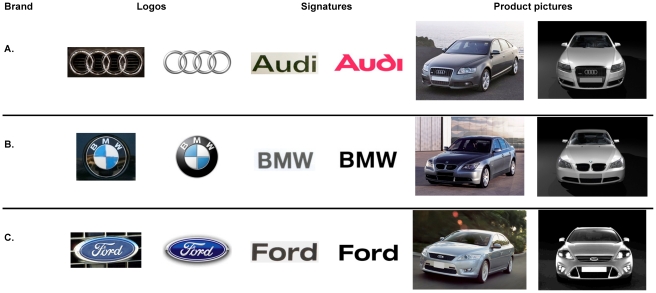
Images used to represent the brands AUDI, BMW, and FORD, varying according to the factor BRAND CUE.

Additionally, stimuli also varied according to another factor—called *brand cue* (through stimuli varying in their level of abstraction). This added complexity in the manipulation served the purpose of further testing the validity of the *md*-IAT. Based on previous findings that identified the IAT to be mostly driven by the category labels and less so by the actual stimuli in the sorting task [12], [27], [29], we expected minor or no differences at all between the different levels of the factor brand cue. The following brand cues served as stimuli for the target categories AUDI and FORD: images of the AUDI/FORD *logo*, images of the AUDI/FORD *signature*, and images of the *products* themselves (i.e., current car models of AUDI/FORD). See figure 1 for target category stimuli used to represent the brands AUDI and FORD. Two stimuli were used to represent each brand (i.e., each target category): a realistic image and an artificial image. Realistic images included real photographs of the logo, the signature, or a specific product model. Artificial images were digitized versions of either the logo or the signature as used in advertising and public relations or simply renderings from computer-aided design drawings of the same product models.

All stimuli for the IATs, both words and pictures, were selected in accordance with suggestions by Nosek and colleagues [27], [40]: First, only stimuli that were clearly and unambiguously associated with a category (or concept) were selected from free association protocols in a pretest. This is a necessary prerequisite to prevent cross category associations from exerting an influence on task performance [27], [41]. For example, it would be impracticable in a race-related IAT to have a stimulus depicting a person with ambiguous face race markers; clearly, this could cause subjects to sort such a stimulus arbitrarily to either category, or to refuse giving a response entirely. Second, a minimum of two stimulus items per target category and three items per attribute category was used throughout the experiment. Previous research showed that the magnitude of IAT effects, reliability, and correlations with direct measures remained stable for IATs assigning two or more stimulus exemplars per category (cf. [40], for results on IATs using 1, 2, 4, 6 or 8 items per category).

#### Apparatus

The various IATs were administered using PsyScope X (build 46) experimental software [42]—both to present the stimuli and to collect the data. The experiment was run on two identically configured Apple Mac mini computers (1.25 GHz PowerPC G4 chip set, 512 MB RAM) with preinstalled OS X v10.4 (Tiger). Participants sat approximately at a distance of 50–55 cm away from the screen—a 19" BenQ FP93V LCD monitor at a resolution of 1280×1024 pixels with a refresh-rate of 75 Hz. Additionally, a USB button box by ioLab served as the default input device, limiting the inaccuracy in measuring reaction times to < = 1.0 ms.

#### General Procedure and Design

The entire experiment required subjects to complete eighteen IATs and a subsequent questionnaire. Data were gathered in three separate test sessions (T1, T2, and T3). The minimum time interval between two sessions was one day. Subjects completed one *md*-IAT (six IATs) per session, one for each of the six bipolar attribute dimensions, taking them approximately 20–35 minutes. All attribute dimensions were in fixed order throughout the entire experiment: (1) safe–unsafe (2) young–old (3) reliable–unreliable (4) aggressive–peaceful (5) environmentally friendly–non-environmentally friendly (6) innovative–conventional. The three dimensions of the factor brand cue (logo, signature, and product) were counterbalanced across subjects. This was necessary as learning effects could be an issue after several administrations of an IAT. Previous research found the magnitude of IAT effects declining for subjects with prior experience. Yet, this was primarily the case for subjects who had previously completed no more than two IATs (see [43]). Little or no further decrease was observed for subjects that had completed more than two IATs [35]. Hence, counterbalancing for brand cue also helped minimizing order effects for the factor attribute dimension. After participants had completed the six IATs at T3, they were prompted to fill out a questionnaire, which also included 7-point semantic differential scales as a direct (or explicit) measure of brand attitudes [30]. The semantic differential scales required subjects to rate each brand separately on the same six attribute dimensions also used for the IATs. Half of the subjects first rated AUDI followed by FORD (vice versa for the other half) to control for order effects.

Written consent was acquired from each participant prior to the experimental sessions. As this was a non-clinical study without any harming procedure and as all data were collected anonymously, ethical approval was not sought for the execution of this study.

#### Procedure and Design of the md-IATs

Following the IAT procedure outlined earlier, the IATs used for the present research were based on the same structure. Each IAT consisted of seven blocks (B1 through B7). Although B3 and B4, and similarly, B6 and B7, were in fact separate blocks, they essentially can be considered one task. There are two reasons for this: first, B3 and B4, and B6 and B7, were identical except for the number of trials used in each block. The number of trials in B3 and B6, and B4 and B7, was 23 and 40, respectively. Second, Greenwald et al. [35] suggested using their new scoring algorithm, the *D measure*, which involves joint analysis of the data in B3 & B4, and also B6 & B7. Other scoring algorithms do not make use of the data in B3 and B6—for the most part, because these blocks were initially devised as practice blocks for the ensuing combined tasks.

Each trial in every block involved subjects sorting just one stimulus, either a word or a picture, to its designated category. The stimuli were presented in the middle of the screen. Each stimulus remained until the subject hit the correct button on the button box. If a subject pressed the wrong button, a red capital X served as error feedback, upon which a subject had to press the other button as fast as possible. The inter-trial interval (ITI), that is, the interval between a correct response to a stimulus and the next stimulus onset was set to 200 ms. Stimuli within the seven blocks were fully randomized, the only restriction being that for the combined tasks a target category stimulus was never followed by another target category stimulus, instead it was always followed by an attribute category stimulus (or vice versa). Finally yet importantly, extraneous effects of task order of the two combined tasks (B3 & B4, B6 & B7) were counterbalanced by two means. First, the display of the target categories (whether AUDI or FORD was first assigned to the left key) was counterbalanced: half of the subjects started with AUDI assigned to the left key and FORD assigned to the right key (vice versa for the other half). For both groups, key assignments for the target categories changed after the initial combined task, with AUDI being assigned to the right key and FORD being assigned to the left key (again vice versa for the other half). Second, the reversed target discrimination task (B5) involved some extra trials in order to provide subjects with the opportunity and the time to unlearn the previous key assignments, and consequently, to learn the new assignments. Nosek, Greenwald, and Banaji [40] provided ample empirical evidence that adding extra trials to the reversed target discrimination task virtually eliminates this unwanted effect of task order. Messner and Vosgerau [44] have recently introduced a new procedure of neutralizing this task order effect by adding iterations of the initial combined task and the reversed combined task to the procedure. This adaptation effectively counteracted the impact of cognitive inertia (i.e., the difficulty in switching between the two tasks) even on the individual level (as opposed to the aggregate level).

### Study 2

Study 2 was intended to replicate the findings of Study 1 with a different set of brands in the *md*-IAT. BMW was chosen to replace FORD as the contrasting brand in the comparisons with AUDI. AUDI and BMW are commonly perceived to be highly similar in terms of several key aspects associated with the brand: for example, in ratings of safety, build quality, reliability, and technical innovativeness [45]. Finding reliable differences between these two highly similar brands (i.e., IAT effects of comparable magnitude across the three levels of the factor brand cue) would provide not just evidence of the *md*-IAT’s reliability but also of its sensitivity. It is evident that finding differences between two highly similar attitude objects asks for a more sensitive measure. Together, Study 1 and Study 2 allow for an assessment of the *md*-IAT procedure and its methodological appropriateness based on its sensitivity, reliability and validity.

#### Participants

Thirty students from the University of Vienna (15 women) participated in the study. Among them a total of 27 received extra undergraduate course credit in return; the remaining three subjects were not associated with the Faculty of Psychology and therefore did not receive anything in exchange. One male subject was excluded due to an unspecified mental condition that impaired his speech and motor behavior. The median age of the remaining twenty-nine subjects (ranging from age 18 to 34) was 22.0 years. An additional three subjects (one woman, two men) were excluded after the analysis of the reaction time data because of an average total error rate of more than 10% across all IATs. Among the remaining participants 26.9% (7) were car-owners. Overall interest in cars was assessed by the same six yes–no questions as in Study 1 and showed to be low among the participants (*M* = 1.65, *SD* = 1.70). All subjects had normal or corrected-to-normal vision (visual acuity was checked with standard Snellen charts).

#### Materials

The materials used for Study 2 were identical to the materials used in Study 1, except for the stimuli related to the new target category brand FORD, which were replaced with stimuli related to BMW (see figure 1). As in the previous study, brand associations were measured on the same six bipolar attribute dimensions. Each pole (or attribute category) was represented by the same three word stimuli.

#### General Procedure and Design

The procedure and design of Study 2 was identical to that of Study 1.

#### Procedure and design of the md-IATs

The procedure and design of the md-IATs was identical to that of Study 1.

## Results

### Study 1

#### Data preparation

As noted earlier, IAT effects are based on differences in reaction times between two experimental tasks: the initial combined task(s) (B3 & B4) and the reversed combined task(s) (B6 & B7). This difference, however, may be computed in different ways. Earlier studies were based on an algorithm that involved dropping the first two trials of each block, discarding subjects‘ trials with responses either below 300 ms or above 3,000 ms—and ultimately, log-transforming the resulting values before computing the IAT-effect by subtracting the averaged log-transformed values of B4 from B7. Recently, Greenwald et al. [35] introduced a new scoring algorithm, the *D measure*, which has since then been adopted by most researchers [22], [46], [47]–[50]. Lane, Banaji, Nosek, and Greenwald [51] recommended the new algorithm, as it proved to be superior to the conventional algorithm in minimizing: (1) the correlation between IAT effects and individual subjects‘ average response latencies, (2) the effect of the order of the IAT blocks, and (3) the effect of previously completing one or more IATs on IAT scores, while (4) retaining strong internal consistency and (5) maximizing the correlation between implicit and explicit measures. The present research opted for a variant of the new scoring algorithm that differed exclusively in terms of its outlier treatment. Instead of using an absolute outlier criterion—dropping trials above 10,000 ms as suggested by Greenwald et al. [35]—boundaries for outliers were set dynamically. For each individual on each of the 18 IATs, trials outside the boundary defined by the mean response latency + 2.5 *SD*s (standard deviations) were excluded from further analysis following the advice of Carbon and Leder [52]. Table 2 gives step-by-step instructions for the *adapted D measure* algorithm. All of the results reported further below are based on the *adapted D measure*. Note: analyses relying on the regular *D measure* (without the dynamic outlier criteria) yielded similar results.

**Table 2:** Adapted D measure algorithm relying on the dynamic outlier criterion.

STEP	ADAPTED D MEASURE ALGORITHM
1	Include trials from B3, B4, B6, B8 in analysis
2	Compute mean latency and standard deviations for each individual and each IAT separately
3	Compute boundary values by adding 2.5 *SDs* to the mean latency
4	Delete all trials above the ‘mean + 2.5 *SD* threshold’
5	Delete subjects with more than 10% of trials below 300 ms
6	No further trials dropped from here (keeping also the first two trials in each block)
7	Compute mean for correct responses for B3, B4, B6, B7
8	Compute one pooled *SD* for all correct responses in B3 & B6; another one for B4 & B7
9	Compute two difference scores: B6 – B3 and B7 – B4
10	Divide each difference by its associated pooled *SD* from step 8
11	Compute the equal-weight average of the two quotients in step 10

*Note.* B3, B4, B6, B7 refer to the different blocks in the IAT scripts. *SD*  =  standard deviation. IAT  =  Implicit Association Test.

#### Main Results

The experiment was based on a 6×3 (*attribute dimension* x *brand cue*) within-subjects design. Table 3 lists all of the 18 IATs in each factor combination, providing both weighted means in milliseconds and means according to the *adapted D measure* along with their standard deviations (*SD*) and effect sizes (*d*). The *adapted D measure* served as input for the statistical analyses. The average effect size across all 18 IATs amounted to *d* = .34.

**Table 3:** Study 1: Summary of all 18 single IATs with factors ATTRIBUTE DIMENSION and BRAND CUE (6×3).

		BRAND CUE											
		*md*-IAT (logos)				*md*-IAT (signatures)				*md*-IAT (product pictures)			
ATTRIBUTE DIMENSION	*N*	*M_ms_*	*M*	*SD*	*d*	*M_ms_*	*M*	*SD*	*d*	*M_ms_*	*M*	*SD*	*d*
1 safe–unsafe	26	8.2	.05	.34	.16	1.0	.06	.41	.15	11.4	.08	.28	.27
2 young–old	26	−14.1	−.15	.45	−.33	−6.9	−.12	.43	−.28	−1.6	−.03	.39	−.09
3 reliable–unreliable	26	15.1	.12	.30	.39	7.8	.10	.37	.29	7.9	.07	.51	.13
4 aggressive–peaceful	26	26.8	.37	.46	.80	15.6	.21	.60	.35	27.0	.35	.51	.68
5 environmental–non-environmental	26	−16.9	−.30	.39	−.78	3.0	−.03	.34	−.09	−14.5	−.17	.26	−.64
6 innovative–conventional	26	−6.2	−.15	.36	−.42	−9.8	−.09	.44	−.22	8.5	.02	.36	.05

*Note. N* indicates number of participants per *md*-IAT; *M_ms_*  =  weighted mean in milliseconds; *M*  =  mean according to participants' *D measure* scores; *SD*  =  standard deviation of the *D measure* scores; effect size measure *d* = *M*/*SD*. Data of this table were processed on basis of the dynamic outlier criterion described above.

**Figure 2:**
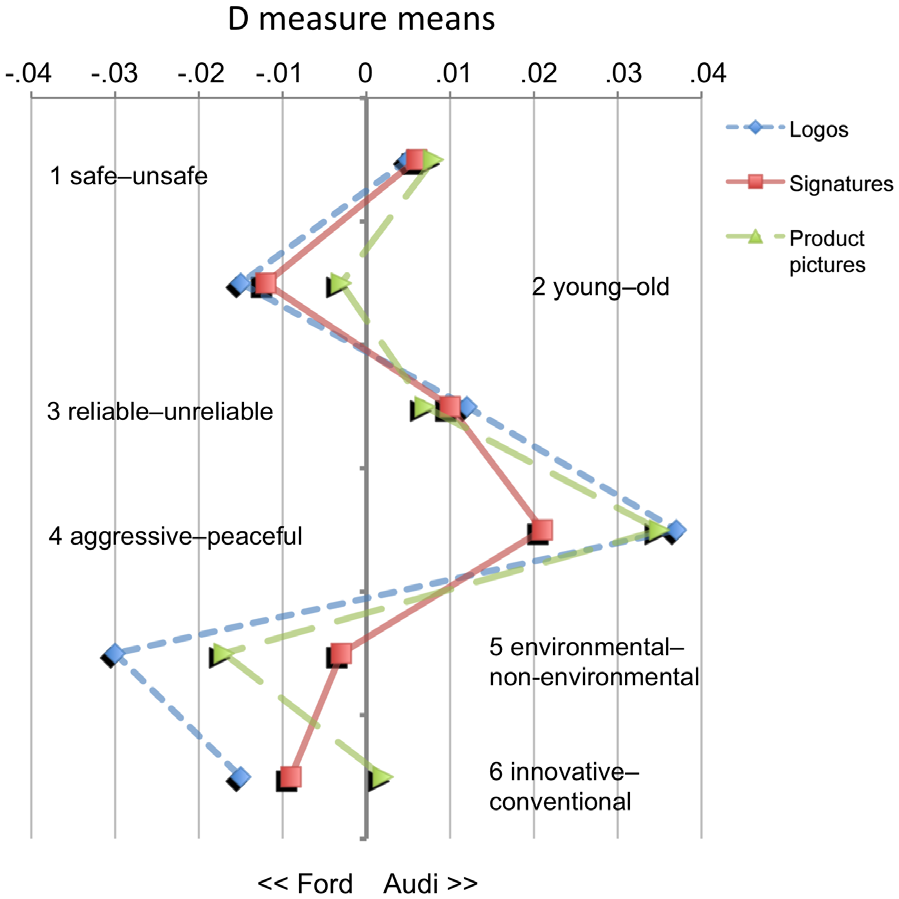
Study 1 (“AUDI vs. FORD”): D measure means for every single IAT (*N* = 26) resulting from combinations of the two factors ATTRIBUTE DIMENSION and BRAND CUE.

As mentioned above, two participants had to be excluded because of an above average overall error rate exceeding 10% of total trials. A repeated-measures ANOVA with the two within-subjects variables *attribute dimension* (i.e., (1) safe–unsafe, (2) young–old (3) reliable–unreliable (4) aggressive–peaceful (5) environmentally friendly–non-environmentally friendly (6) innovative–conventional) and *brand cue* (i.e., logo, signature, product) revealed a main effect of attribute dimension, *F_GG_*(2.60, 64.94) = 7.98, *p*<.001, *η*_p_^2^ = .24 (corrected for Greenhouse-Geisser). Mauchly's test of sphericity showed that the assumption of sphericity had been violated, *X^2^*(14), *p*<.001; degrees of freedom were corrected according to Greenhouse-Geisser estimates of sphericity (ε = .52). All *F*-values missing the subscript “*GG*” were not corrected. This main effect, however, was not relevant for the objectives of the present research. Differences for the factor attribute dimension were expected, simply because each dimension was intended to measure unique aspects of the overall attitude. As expected, there was no main effect observed for the other factor, brand cue, *F*(1.51, 37.77) = 1.05, *p* = .34, *ns.* Likewise, we did not find an interaction between *attribute dimension* and *brand cue*, *F*(10, 250) = 1.68, *p* = .09, *ns.* Figure 2 shows that the variable brand cue only accounted for relatively minor variations within each of the six attribute dimensions.

#### Reliability and Validity of the md-IAT

To calculate split-half reliabilities for each of the 18 IATs in the *md*-IAT, we followed the procedure by De Houwer and De Bruycker [22]. For each IAT, we first listed all the trials by order of appearance, separately for each stimulus type (AUDI, FORD, positive, negative), test block (AUDI-positive, FORD-positive) and participant. Following this, separate IAT-effects (operationalized by the *adapted D measure*) were calculated for odd and even subsets of those trial-response lists. The average split-half reliability in Study 1 was *r* = .79, *SD* = .13. Table 4 provides the split-half reliabilities for all of the 18 IATs.

**Table 4:** Split-half estimates of reliability for each of the 6×3 IATs in Study 1 and Study 2.

		BRAND CUE					
		*md*-IAT (logos)		*md*-IAT (signatures)		*md*-IAT (product pictures)	
		Study 1	Study 2	Study 1	Study 2	Study 1	Study 2
ATTRIBUTE DIMENSION	*N*	*r*	*r*	*r*	*r*	*r*	*r*
1 safe–unsafe	26	.72	.79	.94	.86	.59	.89
2 young–old	26	.89	.86	.91	.87	.67	.87
3 reliable–unreliable	26	.64	.71	.83	.73	.87	.71
4 aggressive–peaceful	26	.73	.86	.92	.89	.85	.82
5 environmental–non-environmental	26	.83	.85	.77	.70	.48	.57
6 innovative–conventional	26	.86	.78	.88	.78	.84	.75

*Note. r* refers to the split-half correlations and describe the reliability (stability) of the extended *md*-IAT procedure. Reliabilities were calculated based on an odd–even split of the trial-responses, following the procedure by De Houwer and De Bruycker [22].

To obtain estimates of the *md*-IAT's convergent validity we compared the results from the IATs to direct (or explicit) ratings of the same six attribute dimensions. The relationship between indirect and direct measures was assessed by several linear regressions—one for each of the six attribute dimensions. IAT-effects were averaged across the three levels of the factor *brand cue* (following the non-significant main effect in the ANOVA) and subsequently compared to direct measures. Based on previous meta-analyses [53], [54], relationships were expected to be positive, varying in magnitude due to factors such as social desirability or ability to introspect. Therefore, all of the *p*-values reported in Table 5 are based on one-tailed tests of significance.

**Table 5:** Study 1: Estimates of convergent validity (simple linear regressions for all six dimensions).

ATTRIBUTE DIMENSION	*N*	*R* = *β*	*R^2^*	*t*(24)	*p*
1 safe–unsafe	26	.494	.244	2.72	.006
2 young–old	26	.242	.058	1.19	.122
3 reliable–unreliable	26	.002	.000	.009	.496
4 aggressive–peaceful	26	.226	.051	1.11	.139
5 environmental–non-environmental	26	.258	.066	1.28	.107
6 innovative–conventional	26	.408	.166	2.14	.022

*Note. p*-values for one-tailed testing.

Besides the main interest in the present study to develop and evaluate the *md*-IAT as an attitudinal, multi-dimensional measure of brand associations, we gained interesting information about the two brands. Ratings derived from the semantic differentials were converted into a difference score in order to make them comparable to the IAT-effect scores. Averaged across the three levels of the factor *brand cue*, the results showed a small effect for the dimensions young–old (*M* = −.10, *SD* = .42, *d* = −.23), reliable–unreliable (*M* = .09, *SD* = .39, *d* = .27), and innovative–conventional (*M* = −.07, *SD* = .39, *d* = −.20)—with FORD being stronger associated with “young” and “innovative” and AUDI being stronger associated with “reliable”. In addition, the results showed a medium effect for the dimensions aggressive–peaceful (*M* = .31, *SD* = .52, *d* = .61) and environmental–non-environmental (*M* = −.17, *SD* = .33, *d* = −.50), with AUDI being stronger associated with “aggressive” and FORD being stronger associated with “environmental”. According to Cohen [55] absolute effect sizes are classified as small, medium, and large, for the following values, *d* = 20, *d* = 50, *d* = 80, respectively.

### Study 2

#### Data preparation

Study 2 utilized the same algorithm as Study 1.

#### Main Results

**Figure 3:**
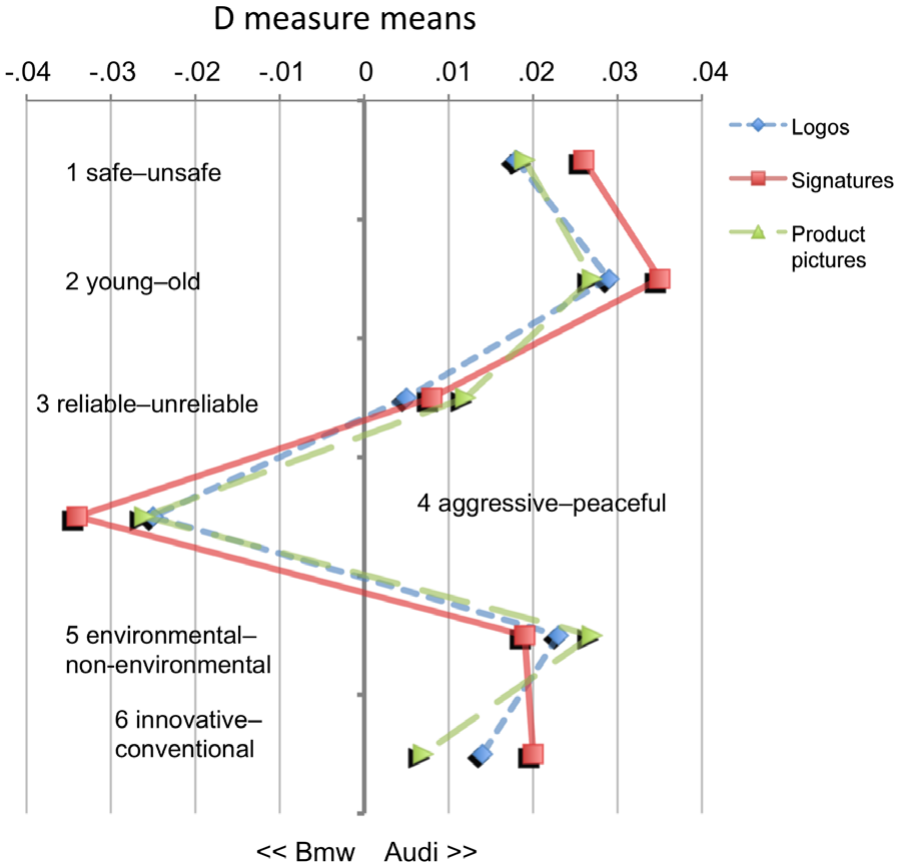
Study 2 (“AUDI vs. BMW”): D measure means for every single IAT (*N* = 26) resulting from combinations of the two factors ATTRIBUTE DIMENSION and BRAND CUE.

The experiment was based on a 6×3 (*attribute dimension* x *brand cue*) within-subjects design. Table 6 lists all of the 18 IATs in each factor combination, providing both weighted means in milliseconds and means according to the *adapted D measure*, along with their standard deviations and effect sizes. The average effect size across all 18 IATs amounted to *d* = .51. A repeated-measures ANOVA revealed a main effect of attribute dimension, *F_GG_ (2.56, 64.08)*  = *10.31, p*<.*001, η_p_* ^2^ = .*29).* Mauchly's test showed that the assumption of sphericity had been violated, *X^2^*(14) = 42.82, *p*<.001; degrees of freedom were corrected according to Greenhouse-Geisser estimates of sphericity (ε = .51). This main effect, however, was not relevant for the objectives of the present research. As in Study 1, differences for the factor attribute dimension were irrelevant (and partly expected), simply because each dimension was intended to measure unique aspects of the overall attitude. As expected, there was no main effect observed for the other factor, brand cue, *F(2, 50)*<1, *p* = .*88*, *ns.* Likewise, we did not find an interaction between attribute dimension and brand cue, *F(10, 250)*<1, *p* = .*60*, *ns.* Similar to Study 1, figure 3 shows that the factor brand cue accounted only for relatively minor variations within each of the six attribute dimensions. Differences were a bit larger for the attribute dimensions 4 (aggressive–peaceful) and 5 (environmental–non-environmental).

**Table 6:** Study 2: Summary of all 18 single IATs with factors ATTRIBUTE DIMENSION and BRAND CUE (6×3).

		BRAND CUE											
		*md*-IAT (logos)				*md*-IAT (signatures)				*md*-IAT (product pictures)			
ATTRIBUTE DIMENSION	*N*	*M_ms_*	*M*	*SD*	*d*	*M_ms_*	*M*	*SD*	*d*	*M_ms_*	*M*	*SD*	*d*
1 safe–unsafe	26	18.9	.18	.40	.44	23.8	.26	.41	.64	20.1	.19	.56	.34
2 young–old	26	24.7	.29	.41	.72	27.3	.35	.49	.71	23.8	.27	.45	.60
3 reliable–unreliable	26	5.0	.05	.35	.13	10.7	.08	.33	.24	13.2	.12	.36	.33
4 aggressive–peaceful	26	−19.9	−.25	.54	−.46	−29.2	−.34	.59	−.59	−18.7	−.26	.41	−.63
5 environmental–non-environmental	26	16.3	.23	.38	.61	12.6	.19	.35	.54	21.0	.27	.33	.83
6 innovative–conventional	26	11.0	.14	.36	.38	13.0	.20	.26	.77	4.4	.07	.38	.18

*Note. N* indicates number of participants per *md*-IAT; *M_ms_*  =  weighted mean in milliseconds; *M*  =  mean according to participants' *D measure* scores; *SD*  =  standard deviation of the *D measure* scores; effect size measure *d* = *M*/*SD.* Data of this table were processed on basis of the dynamic outlier criterion described above.

#### Reliability and Validity of the md-IAT

As in Study 1, reliabilities were calculated based on an odd–even split of the trial-responses, following the procedure by De Houwer and De Bruycker [22]. The average split-half reliability in Study 2 was *r* = .79, *SD* = .09. Again, refer to table 4 for the split-half reliabilities for all of the 18 IATs.

Estimates of the *md*-IAT's convergent validity were obtained by comparing the results from the IATs to direct (or explicit) ratings of the same six attribute dimensions following the same procedure as in Study 1. Table 7 below shows the results of the regression analyses.

**Table 7:** Study 2: Estimates of convergent validity (simple linear regressions for all six dimensions).

ATTRIBUTE DIMENSION	*N*	*R* = *β*	*R^2^*	*t*(24)	*p*
1 safe–unsafe	26	.301	.090	1.55	.068
2 young–old	26	.419	.176	2.26	.017
3 reliable–unreliable	26	.093	.009	.456	.326
4 aggressive–peaceful	26	.345	.119	1.80	.043
5 environmental–non-environmental	26	.232	.054	1.17	.127
6 innovative–conventional	26	.462	.213	2.55	.009

*Note. p*-values for one-tailed testing.

As in Study 1 the results of the *md*-IAT also revealed interesting information about consumers' brand associations for the two brands. Averaged across the three levels of the factor *brand cue*, the results showed a small effect for the dimensions safe–unsafe (*M* = .21, *SD* = .46, *d* = .47), reliable–unreliable (*M* = .08, *SD* = .35, *d* = .23) and innovative–conventional (*M* = .14, *SD* = .33, *d* = .44)—with AUDI being stronger associated with the attributes “safe”, “reliable”, and “innovative”. In addition, the results showed a medium effect for the dimensions young–old (*M* = .30, *SD* = .45, *d* = .68), aggressive–peaceful (*M* = −.28, *SD* = .51, *d* = −.56) and environmental–non-environmental (*M* = .23, *SD* = .35, *d* = .66)—with AUDI being stronger associated with “young” and “environmental” and BMW being stronger associated with “aggressive”.

## Discussion

With the Implicit Association Test Greenwald et al. [1] have radically innovated research on attitudes in general. Over the last decade the IAT has become the most popular indirect measure of attitudes, welcomed by researchers and marketing practitioners alike as a tool to measure attitudes in a rather indirect and implicit way, unlike common explicit measures such as verbal self-reports. The IAT is deemed to be a promising alternative, particularly for measuring attitudes consumers may not be aware of, able to express, or willing to share with the researcher [2], [3]. The *multi-dimensional* Implicit Association Test (*md*-IAT) constitutes an extension of the IAT procedure that goes beyond measuring attitudes on a single dimension only (e.g., good–bad); that is, with the *md*-IAT it is possible to measure different nuances of a global attitude (e.g., on scales such as safe–unsafe; young–old; innovative–conventional; etc.). As a consequence, the *md*-IAT procedure (i.e., multiple measurement on more than just one attribute dimension) yields a more detailed representation of consumers' evaluations of a brand or product. Being of high practical relevance, this gain in dimensionality provides more insight and therefore more opportunities for specific intervention.

The results of Study 1 (“AUDI/FORD”) and Study 2 (“AUDI/BMW”) provide strong evidence of the *md*-IAT's methodological appropriateness. Split-half reliabilities averaged *r* = .79 (n = 2×18 IATs) for both studies. For comparison, in a meta-analysis Hofmann et al. [26] reported the same mean reliability of *r* = .79 (*n* = 50) for the IAT. Regarding the *md*-IATs convergent validity, regression analyses of the six *md*-IAT dimensions and the direct measures revealed that, except for one dimension (reliable–unreliable), *R*-values (for simple regressions *R*-values are identical with the correlation coefficients) were of close to average or above average magnitude: Hofmann et al. [26] reported an average indirect–direct correlation of .34 for consumer research related studies (based on *n* = 11 independent studies). Considering this meta-analytic finding, the results of the present studies fit well into the overall picture.

As a test of external validity we varied stimuli in the perceptual domain (i.e., through the three levels of the factor *brand cue*: logo, signature, product). In both studies, the factor *brand cue* was not significant and therefore accounted only for minor variations of the *adapted D measure* means within each of the six attribute dimensions. These results show that the *md*-IAT can be rather seen as a conceptual measure of brand associations—widely unaffected by perceived stimulus variations (characteristics) in the perceptual domain of a brand [56]. While this is—in most cases—viewed as an advantage, the *md*-IAT is therefore less suited for testing the impact of specific (product) designs (e.g., visual identifiers) on brand associations. As a last indicator of methodological appropriateness, sensitivity of the *md*-IAT can be regarded as reasonable. Despite the fact that the two brands used in Study 2 (“AUDI/BMW”) are commonly perceived to be highly similar, which could make finding differences difficult, we did not find any decrease in sensitivity compared to the two brands used in Study 1 (“AUDI/FORD”). On the contrary, effect sizes averaged *d* = .34 in Study 1 and *d* = .51 in Study 2 across all 6×3 IATs part of the *md*-IAT, indicating a small average and a medium average effect, respectively.

Finally, brand attitudes as revealed by the *md*-IAT indicate that FORD is judged “slightly younger”, “slightly more innovative”, “more environmental”, “less aggressive”, but also “less reliable” than AUDI. Study 2 revealed that AUDI is judged as “slightly safer”, “slightly more reliable”, “slightly more innovative”, “younger”, “more environmental”, and “less aggressive” than BMW.

### Conclusions

Based on the results of the present research, the *multi-dimensional* Implicit Association Test (*md*-IAT) has shown to be a reliable, valid, and sensitive indirect measure of brand attitudes. Regular one-dimensional IATs are useful if one is only interested in an overall brand attitude (e.g., are people's attitudes more favorable toward AUDI or to BMW?). The main advantage of the *md*-IAT lies in its more detailed, multi-dimensional assessment. Marketing practitioners in particular might value the additional information offered by the *md*-IAT, for example allowing them to easily create complex and differentiated brand profiles, and thus distinguishing between different components of an overall brand attitude (i.e., tapping into the multifaceted nature of consumers' brand associations). Similarly, academics might find the *md*-IAT useful for testing constructs such as brand or product personality [9], [10] also with indirect measures. Just as the IAT, its *multi-dimensional* extension (*md*-IAT) is better suited for measuring attitudes consumers are not consciously aware of, able to express, or willing to share with the researcher [2].

## References

[1] Greenwald AG, McGhee DE, Schwartz JLK (1998). Measuring individual differences in implicit cognition: The implicit association test.. Journal of Personality and Social Psychology.

[2] Brunel FF, Tietje BC, Greenwald AG (2004). Is the Implicit Association Test a valid and valuable measure of implicit consumer social cognition?. Journal of Consumer Psychology.

[3] Friese M, Wanke M, Plessner H (2006). Implicit consumer preferences and their influence on product choice.. Psychology and Marketing.

[4] Rudman LA, Kilianski SE (2000). Implicit and explicit attitudes toward female authority.. Personality and Social Psychology Bulletin.

[5] Egloff B, Schmukle SC (2002). Predictive validity of an implicit association test for assessing anxiety.. Journal of Personality and Social Psychology.

[6] Gray NS, Brown AS, MacCulloch MJ, Smith J, Snowden RJ (2005). An implicit test of the associations between children and sex in pedophiles.. Journal of Abnormal Psychology.

[7] Faerber SJ, Leder H, Gerger G, Carbon C-C (2010). Priming semantic concepts affects the dynamics of aesthetic appreciation..

[8] Keller KL (2003). Brand synthesis: The multidimensionality of brand knowledge.. Journal of Consumer Research.

[9] Govers PCM, Schoormans JPL (2005). Product personality and its influence on consumer preference.. The Journal of Consumer Marketing.

[10] Aaker JL (1997). Dimensions of brand personality.. Journal of Marketing Research.

[11] Saaksjarvi M, Samiee S (2007). Nonprice antecedents of consumer preference for cyber and extension brands.. Journal of Interactive Marketing.

[12] Fazio RH, Olson MA (2003). Implicit measures in social cognition research: Their meaning and uses.. Annual Review of Psychology.

[13] Carbon C-C, Michael L, Leder H (2008). Design evaluation by combination of repeated evaluation technique and measurement of electrodermal activity.. Research in Engineering Design.

[14] Carbon C-C, Hutzler F, Minge M (2006). Innovativeness in design investigated by eye movements and pupillometry.. Psychology Science.

[15] Windhager S, Hutzler F, Carbon C-C, Oberzaucher E, Schaefer K (2010). Laying eyes on headlights: Eye tracking reveals facial features in cars..

[16] Hess U, Sabourin G, Kleck RE (2007). Postauricular and eyeblink startle responses to facial expressions.. Psychophysiology.

[17] McClure SM, Li J, Tomlin D, Cypert KS, Montague LM (2004). Neural correlates of behavioral preference for culturally familiar drinks.. Neuron.

[18] Cunningham WA, Zelazo PD (2007). Attitudes and evaluations: a social cognitive neuroscience perspective.. Trends in Cognitive Sciences.

[19] Fazio RH, Sanbonmatsu DM, Powell MC, Kardes FR (1986). On the automatic activation of attitudes.. Journal of Personality and Social Psychology.

[20] De Houwer J (2003). The extrinsic affective Simon task.. Experimental Psychology.

[21] Nosek BA, Banaji MR (2001). The Go/No-go Association Task.. Social Cognition.

[22] De Houwer J, De Bruycker E (2007). The implicit association test outperforms the extrinsic affective Simon task as an implicit measure of inter-individual differences in attitudes.. British Journal of Social Psychology.

[23] Nosek BA (2007). Implicit - explicit relations.. Current Directions in Psychological Science.

[24] Maison D, Greenwald AG, Bruin RH (2004). Predictive validity of the Implicit Association Test in studies of brands, consumer attitudes, and behavior.. Journal of Consumer Psychology.

[25] Nosek BA, Banaji MR, Greenwald AG (2002). Math  =  male, me  =  female, therefore math not-equal-to me.. Journal of Personality and Social Psychology.

[26] Hofmann W, Gawronski B, Gschwendner T, Le H, Schmitt M (2005). A meta-analysis on the correlation between the implicit association test and explicit self-report measures.. Personality and Social Psychology Bulletin.

[27] Nosek BA, Greenwald AG, Banaji MR (2007). The Implicit Association Test at age 7: A methodological and conceptual review: Psychology Press.

[28] Schnabel K, Asendorpf JB, Greenwald AG (2008). Assessment of individual differences in implicit cognition A review of IAT measures.. European Journal of Psychological Assessment.

[29] De Houwer J (2001). A structural and process analysis of the implicit association test.. Journal of Experimental Social Psychology.

[30] Osgood CE, Suci GJ, Tannenbaum PH (1957). The measurement of meaning..

[31] Eagly AH, Chaiken S (1993). The psychology of attitudes..

[32] Likert R (1932). A technique for the measurement of attitudes.. Archives of Psychology.

[33] Greenwald AG, Banaji MR (1995). Implicit social cognition: Attitudes, self-esteem, and stereotypes.. Psychological Review.

[34] Gawronski B, Hofmann W, Wilbur CJ (2006). Are "implicit" attitudes unconscious?. Consciousness and Cognition.

[35] Greenwald AG, Nosek BA, Banaji MR (2003). Understanding and using the Implicit Association Test: I. An improved scoring algorithm.. Journal of Personality and Social Psychology.

[36] Fishbein M, Ajzen I (1975). Belief, attitude, intention, and behavior: an introduction to theory and research..

[37] Leder H, Carbon C-C (2005). Dimensions in appreciation of car interior design.. Applied Cognitive Psychology.

[38] Carbon C-C (2010). The cycle of preference: Long-term dynamics of aesthetic appreciation.. Acta Psychologica.

[39] Carbon C-C, Leder H (2005). The Repeated Evaluation Technique (RET). A Method to Capture Dynamic Effects of Innovativeness and Attractiveness.. Applied Cognitive Psychology.

[40] Nosek BA, Greenwald AG, Banaji MR (2005). Understanding and using the Implicit Association Test: II. Method variables and construct validity.. Personality and Social Psychology Bulletin.

[41] Bluemke M, Friese M (2006). Do features of stimuli influence IAT effects?. Journal of Experimental Social Psychology.

[42] Cohen J, MacWhinney B, Flatt M, Provost J (1993). Psyscope - an Interactive Graphic System for Designing and Controlling Experiments in the Psychology Laboratory Using Macintosh Computers.. Behavior Research Methods Instruments & Computers.

[43] Greenwald AG, Nosek BA (2001). Health of the Implicit Association Test at age 3.. Zeitschrift fur Experimentelle Psychologie.

[44] Messner C, Vosgerau J (2010). Cognitive inertia and the Implicit Association Test.. Journal of Marketing Research.

[45] Katemann J (2007). Dreh-Freude..

[46] Steffens MC, Konig SS (2006). Predicting spontaneous big five behavior with implicit association tests.. European Journal of Psychological Assessment.

[47] Banse R, Kowalick C (2007). Implicit attitudes towards romantic partners predict well-being in stressful life conditions: Evidence from the antenatal maternity ward.. International Journal of Psychology.

[48] Bar-Anan Y, Liberman N, Trope Y (2006). The association between psychological distance and construal level: Evidence from an implicit association test.. Journal of Experimental Psychology-General.

[49] Pinter B, Greenwald AG (2005). Clarifying the Role of the "Other" Category in the Self-Esteem IAT.. Experimental Psychology.

[50] Schröder-Abé M, Rudolph A, Wiesner A, Schütz A (2007). Self-esteem discrepancies and defensive reactions to social feedback.. International Journal of Psychology.

[51] Lane KA, Banaji MR, Nosek BA, Greenwald AG, Schwarz N, Wittenbrink B (2007). Understanding and Using the Implicit Association Test: IV: What We Know (So Far) about the Method.. Implicit measures of attitudes.

[52] Carbon C-C, Leder H (2005). When feature information comes first! Early processing of inverted faces.. Perception.

[53] Hoffman AB, Murphy GL (2006). Category dimensionality and feature knowledge: When more features are learned as easily as fewer.. Journal of Experimental Psychology-Learning Memory and Cognition.

[54] Nosek BA, Smyth FL, Hansen JJ, Devos T, Lindner NM (2007). Pervasiveness and correlates of implicit attitudes and stereotypes.. European Review of Social Psychology.

[55] Cohen J (1992). A power primer.. Psychological Bulletin.

[56] Person O, Snelders D (2010). Brand styles in commercial design.. Design Issues.

